# Screening for pre‐eclampsia using pregnancy‐associated plasma protein‐A or placental growth factor measurements in blood samples collected at 8–14 weeks' gestation

**DOI:** 10.1002/uog.29204

**Published:** 2025-03-24

**Authors:** L. Rode, A. Wright, D. Wright, M. Overgaard, L. Sperling, P. Sandager, P. Nørgaard, F. S. Jørgensen, H. Zingenberg, I. Riishede, A. Tabor, C. K. Ekelund, the PRESIDE Study Group, M. R. Andersen, L. Bathum, C. A. Juel Jensen, C. S. Knudsen, N. G. Pedersen, K. Pihl, C. Vedel, H. Skov, S. R. Wagner

**Affiliations:** ^1^ Department of Clinical Biochemistry, Rigshospitalet Copenhagen University Hospital Copenhagen Denmark; ^2^ Center for Fetal Medicine and Pregnancy, Department of Gynecology, Fertility, and Obstetrics, Rigshospitalet Copenhagen University Hospital Copenhagen Denmark; ^3^ Institute of Health Research University of Exeter Exeter UK; ^4^ Department of Clinical Biochemistry Odense University Hospital Odense Denmark; ^5^ Department of Clinical Research University of Southern Denmark Odense Denmark; ^6^ Fetal Medicine Unit, Department of Obstetrics and Gynecology Odense University Hospital Odense Denmark; ^7^ Department of Obstetrics and Gynecology, Center for Fetal Medicine Aarhus University Hospital Aarhus Denmark; ^8^ Department of Clinical Medicine Aarhus University Aarhus Denmark; ^9^ Center for Fetal Diagnostics Aarhus University Hospital Aarhus Denmark; ^10^ Department of Obstetrics and Gynecology Copenhagen University Hospital North Zealand Hillerød Denmark; ^11^ Fetal Medicine Unit, Department of Obstetrics and Gynecology Copenhagen University Hospital Hvidovre Hvidovre Denmark; ^12^ Department of Clinical Medicine, Faculty of Health and Medical Sciences University of Copenhagen Copenhagen Denmark; ^13^ Fetal Medicine Unit, Department of Obstetrics and Gynecology Copenhagen University Hospital Herlev and Gentofte Herlev Denmark

**Keywords:** competing‐risks model, Fetal Medicine Foundation, first trimester, placental growth factor, pre‐eclampsia, pregnancy‐associated plasma protein‐a, screening

## Abstract

**Objectives:**

To assess the value of pregnancy‐associated plasma protein‐A (PAPP‐A) in screening for preterm pre‐eclampsia (PE) (delivery < 37 weeks' gestation) measured in maternal blood samples collected before 11 weeks, and to compare the screening performance of PAPP‐A with that of placental growth factor (PlGF) from blood samples collected at 8–14 weeks.

**Methods:**

This study analyzed data from women who participated in the PRESIDE (Pre‐eclampsia Screening in Denmark) study, a prospective, non‐interventional multicenter study investigating the predictive performance of the Fetal Medicine Foundation first‐trimester screening algorithm for PE in a Danish population. As part of combined first‐trimester screening, a routine blood sample was collected at 8–14 weeks' gestation and PAPP‐A was measured. Excess serum was stored at −80°C and analyzed for PlGF in batches after delivery. Most women in the PRESIDE study had an extra blood sample collected at the time of the first‐trimester scan at 11–14 weeks, which was also analyzed for PlGF and PAPP‐A in batches after all the participants had delivered. Screening performance was assessed in terms of the detection rate at a 10% screen‐positive rate (SPR) for a combination of PAPP‐A or PlGF with maternal factors alone and for a combination of each of these biomarkers with maternal factors, mean arterial pressure (MAP) and uterine artery pulsatility index (UtA‐PI).

**Results:**

The study population comprised 8386 women who had a routine combined first‐trimester aneuploidy screening blood sample collected at 8–14 weeks' gestation. In pregnancies that developed preterm PE, the median PAPP‐A multiples of the median from routine blood samples were 0.78 (95% CI, 0.67–0.90) before 10 weeks, 0.80 (95% CI, 0.58–1.10) at 10 weeks and 0.64 (95% CI, 0.53–0.78) at 11–14 weeks. In women with samples collected before 10 weeks, there was no significant improvement in the detection rate of preterm PE when PAPP‐A or PlGF was combined with maternal factors alone or when combined with maternal factors, MAP and UtA‐PI. In routine samples collected at or after 10 weeks, PAPP‐A only increased the detection rate of preterm PE slightly. However, PlGF in samples collected at or after 10 weeks increased the detection rate from 31.3% (95% CI, 16.1–50.0%) to 56.3% (95% CI, 37.7–73.6%) at a 10% SPR, i.e. an increase in the detection rate of 25.0% (95% CI, 4.3–44.4%), when combined with maternal factors alone. When PlGF collected from the PRESIDE sample at 11–14 weeks was combined with maternal factors, MAP and UtA‐PI, there was an increase in the detection rate from 50.9% (95% CI, 37.1–64.6%) to 67.3% (95% CI, 53.3–79.3%), i.e. an increase of 16.4% (95% CI, 5.6–29.0%) at a 10% SPR.

**Conclusions:**

PAPP‐A has limited value in first‐trimester screening for PE, whereas PlGF adds significantly to the detection rate of preterm PE at 10–14 weeks' gestation. © 2025 The Author(s). *Ultrasound in Obstetrics & Gynecology* published by John Wiley & Sons Ltd on behalf of International Society of Ultrasound in Obstetrics and Gynecology.

## INTRODUCTION

Blood sampling for combined first‐trimester aneuploidy screening (cFTS) can be conducted from 8 weeks' gestation^1^, ^2^, ^3^, whereas the first‐trimester scan must be performed at 11–14 weeks^4^. The best performance of cFTS, including measurement of pregnancy‐associated plasma protein‐A (PAPP‐A) and free beta‐human chorionic gonadotropin (β‐hCG), is obtained when blood is collected before 11 weeks^3^, ^5^, ^6^, ^7^, ^8^. Early blood sampling enables the use of a two‐step approach, in which blood is collected and analyzed before the first‐trimester scan to ensure that all relevant biomarkers for risk assessment have been obtained by the time of this scan. This two‐step approach is an alternative to the one‐stop clinic for assessment of risk (OSCAR), for which blood samples are drawn on the day of ultrasound assessment, between 11 + 0 and 13 + 6 weeks^4^.

A screening model for pre‐eclampsia (PE) has been developed by the Fetal Medicine Foundation (FMF)^9^, ^10^, ^11^ using biomarkers collected between 11 and 14 weeks' gestation^12^. This screening model detects approximately 75% of preterm PE (delivery before 37 weeks) at a 10% screen‐positive rate (SPR). The model includes maternal factors, mean arterial pressure (MAP), uterine artery pulsatility index (UtA‐PI) and placental growth factor (PlGF), with the option to include PAPP‐A.

Plasma PAPP‐A levels collected at 11–14 weeks are lower in women who will develop PE later in the pregnancy, but there is sparse evidence regarding the predictive performance of PAPP‐A in PE screening when undertaken before 10 weeks^13^, ^14^, ^15^. After 11 weeks, maternal plasma PlGF is a reliable marker for discriminating between pregnancies that will develop and those that will not develop PE. Furthermore, although evidence for its predictive performance before 11 weeks is limited^16^, ^17^, ^18^, ^19^, it has recently been suggested that PlGF may also be useful in screening as early as week 10 of gestation^20^.

In centers and countries using the two‐step approach to first‐trimester screening, it would be advantageous to integrate PAPP‐A in screening for PE. Therefore, the aim of this study was to assess the value of PAPP‐A in screening for PE when measured in maternal blood samples collected routinely before 11 weeks and to compare its screening performance for preterm PE with that of PlGF measured in maternal blood samples collected at various gestational‐age windows from 8 to 14 weeks.

## METHODS

This study analyzed a subgroup of women who participated in the PRESIDE (Pre‐eclampsia Screening in Denmark) study, a prospective, non‐interventional multicenter study investigating the predictive performance of the FMF first‐trimester screening algorithm for PE in a Danish population. Further details of the PRESIDE study have been reported previously^21^. In summary, women with a singleton pregnancy were recruited at the time of cFTS, between May 2019 and December 2020, to the PRESIDE study from six Danish university hospitals (Copenhagen University Hospital Rigshospitalet, Copenhagen University Hospital Herlev, Copenhagen University Hospital Hvidovre, Copenhagen University Hospital North Zealand, Odense University Hospital and Aarhus University Hospital). Women who were younger than 18 years of age, had a multiple pregnancy or were unable to understand Danish or English were excluded. Information on maternal characteristics and on acetylsalicylic acid use was collected via questionnaires that participants filled out at the time of the first‐trimester scan (at 11–14 weeks). All data were entered and stored in the local fetal medicine database (Astraia; Astraia GmbH, Munich, Germany). Information on the use of acetylsalicylic acid among included women was verified by reviewing maternal records. The use of acetylsalicylic acid was based on maternal risk factors, largely informed by the UK National Institute for Health and Care Excellence high‐risk factors, and was recorded in 3% of the included women. Blood pressure was measured in accordance with international guidelines at 11–14 weeks^22^, using an automated blood pressure measurement station that was set up for this study^23^. Measurements of right and left UtA‐PI were performed by pulsed‐wave transabdominal color Doppler at the time of the first‐trimester scan by sonographers who had obtained the FMF certificate of competence in PE screening^24^. Women included in the PRESIDE study gave written informed consent before enrolment, and the study was approved by the Danish Data Protection Agency (P‐2019‐89) and the Research Ethics Committee (H‐19001203).

### Outcomes

As described previously^21^, pregnancy outcomes were collected from birth registries. We validated diagnoses by reviewing the maternal records of women with a diagnosis of PE or preterm birth before 37 weeks and for a random sample of 15% of women without a diagnosis of PE. Outcomes of women with PE were categorized according to gestational age at delivery as preterm PE with delivery before 37 weeks, and term PE with delivery at or after 37 weeks. Gestational age at birth was calculated based on measurements of crown–rump length at the time of cFTS. PE was defined according to the 2018 guidelines of the International Society for the Study of Hypertension in Pregnancy^25^.

### Blood samples and biochemical analyses in PRESIDE study

As part of cFTS, a routine maternal blood sample was collected at 8–14 weeks' gestation. These blood samples were handled routinely and analyzed for PAPP‐A and free β‐hCG. Excess serum was stored at −80°C and analyzed for PlGF in batches, after all women had delivered. Additionally, most women enrolled in the PRESIDE study had an additional blood sample collected at the time of the first‐trimester scan (at 11–14 weeks), regardless of the gestational age at collection of the routine blood sample. Note that it was not mandatory to have the additional blood sample taken in the PRESIDE study, therefore some women included in the present study had only a routine cFTS blood sample. Serum was stored at −80°C and analyzed in batches for PAPP‐A and PlGF, after all women had delivered. All biochemical analyses were performed using the BRAHMS KRYPTOR compact PLUS or KRYPTOR GOLD platforms (BRAHMS GmbH, Hennigsdorf, Germany) at the department of clinical biochemistry at each of the recruiting hospitals.

In the present study, the population consisted of a group of participants from the PRESIDE study, for whom information on maternal demographic characteristics and medical history (referred to hereafter as ‘maternal factors’) and biochemical parameters, including PAPP‐A and PlGF, from the routine cFTS sample and/or the PRESIDE sample were available.

### Statistical analysis

R software version 4.3.2 (R Foundation, Vienna, Austria) was used for all statistical analysis^26^, ^27^. Data were summarized according to whether a diagnosis of PE was registered or not, as median (interquartile range) for continuous variables and *n* (%) for categorical variables.

PAPP‐A multiples of the median (MoMs), with 95% CIs, were grouped by preterm PE before 37 weeks, PE at or after 37 weeks or no PE and plotted against gestational age at blood sampling to assess the discrimination between these three groups by gestational age at sampling. To allow for a direct comparison of the discrimination of early PAPP‐A *vs* PlGF levels in these samples, MoMs were converted into *Z*‐scores and plotted as described above. The FMF competing‐risks model was used to estimate patient‐specific risk of delivery with preterm PE by a combination of maternal demographic characteristics and maternal factors, MAP, UtA‐PI and either PAPP‐A MoM or PlGF MoM. We did not adjust for use of acetylsalicylic acid, as our previous analyses have shown only negligible effects on performance estimates in this population^21^. Women with a missing outcome or those who had a termination of pregnancy were not excluded from the analysis but were classified as unaffected, as the aim was to obtain a clinically applicable conservative intention‐to‐treat analysis of screening performance, and because the anticipated missing data would be about 1%, corresponding to less than one woman developing preterm PE.

The screening performance using PAPP‐A or PlGF from routine samples collected before 10 weeks and at 10–14 weeks was assessed in terms of the detection rate at a 10% SPR, for a combination of PAPP‐A or PlGF with maternal factors alone and for a combination of each of these biomarkers with maternal factors, MAP and UtA‐PI. Correspondingly, the screening performance using one of the two biochemical markers from the PRESIDE samples collected at 11–14 weeks was assessed. In addition, the difference in detection rate with the addition of either PAPP‐A or PlGF was assessed using McNemar's test. *P* < 0.05 was considered statistically significant.

## RESULTS

**Figure 1:**
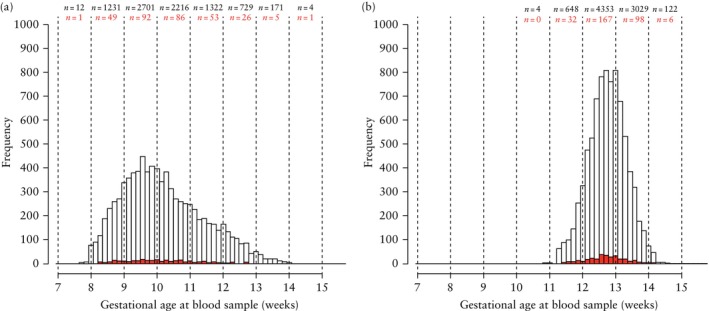
Histogram showing distribution of gestational age at time of routine blood sampling (a) and at time of PRESIDE blood sampling (b). Histogram bars represent number of samples per day (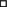) and distribution of blood samples among women who subsequently developed pre‐eclampsia (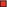). Total number of blood samples collected per week (black text) and number of samples collected from pregnancies affected by pre‐eclampsia (at any gestation) (red text) are given for each gestational week.

The distribution of maternal characteristics of the 8386 women included in this study with available PAPP‐A and PlGF measurements from a routine blood sample collected at cFTS is shown in Table 1. Among these women, 313 (3.7%) developed PE. A total of 7834 (93.4%) women had PAPP‐A and PlGF measurements from the routine cFTS blood sample collected at 8–14 weeks as well as measurements from the PRESIDE blood sample collected at 11–14 weeks. The remaining 552 women had PAPP‐A and PlGF measurements only from the blood sample collected at cFTS (Figure S1). A further 322 women had only a PRESIDE blood sample available, collected at 11–14 weeks, as it was not possible to retrieve their stored blood sample collected at cFTS; these cases were only included in analyses of all women with a PRESIDE sample. The distribution of gestational age at routine cFTS sampling is shown in Figure 1a. The proportion of routine blood samples collected before 11 weeks was 73.5% (*n* = 6160). The distribution of gestational age at PRESIDE sampling is shown in Figure 1b.

**Table 1:** Maternal and pregnancy characteristics in women with and those without pre‐eclampsia (PE) diagnosis among 8386 women with routine blood sample collected at 8–14 weeks' gestation as part of combined first‐trimester screening

Characteristic	Unaffected (*n* = 8073)	PE (*n* = 313)	*P*
Maternal age (years)	30.8 (28.2–33.9)	30.5 (27.6–34.2)	0.66
Maternal weight (kg)	66.0 (60.0–75.1)	70.5 (62.0–82.0)	< 0.001
Maternal height (cm)	168 (164–173)	167 (162–171)	< 0.001
Maternal BMI (kg/m^2^)	23.3 (21.2–26.4)	25.4 (22.3–29.8)	< 0.001
GA at first‐trimester screening (days)	89 (87–92)	89 (86–91)	0.20
Ethnicity			0.90
White	7664 (94.9)	299 (95.5)	
Black	60 (0.7)	3 (1.0)	
East Asian	81 (1.0)	2 (0.6)	
South Asian	153 (1.9)	6 (1.9)	
Mixed	115 (1.4)	3 (1.0)	
Medical history			
Chronic hypertension	29 (0.4)	10 (3.2)	< 0.001
Type‐I DM	4 (0.05)	0 (0)	0.02
Type‐II DM	2 (0.02)	1 (0.3)	0.02
SLE/APS	22 (0.3)	4 (1.3)	0.005
Smoker	246 (3.0)	13 (4.2)	0.35
Family history of PE	274 (3.4)	26 (8.3)	< 0.001
Mode of conception			0.001
Spontaneous	7305 (90.5)	264 (84.3)	
IVF	527 (6.5)	36 (11.5)	
Ovulation drugs	241 (3.0)	13 (4.2)	
Parity			< 0.001
Nulliparous	4241 (52.5)	226 (72.2)	
Parous, no previous PE	3645 (45.2)	54 (17.3)	
Parous, previous PE	187 (2.3)	33 (10.5)	
No delivery outcome available*			NA
Termination before 21 + 6 weeks	13 (0.2)	—	
Miscarriage	18 (0.2)	—	
Intrauterine death ≥ 22 + 0 weeks	2 (0.02)	—	
Delivery at home or at private clinic	19 (0.2)	—	
Moved out of Denmark before delivery	30 (0.4)	—	
Records were non‐accessible	34 (0.4)	—	

Data are given as median (interquartile range) or *n* (%).

* Women for whom delivery outcome was unavailable were treated as not having PE (unaffected) in analyses.

APS, antiphospholipid syndrome; BMI, body mass index; DM, diabetes mellitus; GA, gestational age; IVF, *in‐vitro* fertilization; NA, not applicable; SLE, systemic lupus erythematosus.

**Figure 2:**
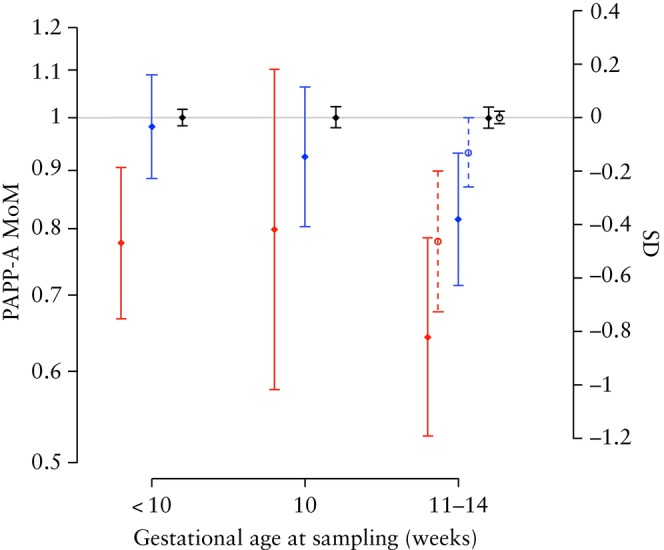
Medians with 95% CIs of pregnancy‐associated plasma protein‐A (PAPP‐A) multiples of the median (MoM) values in pregnancies with pre‐eclampsia (PE) with delivery < 37 weeks (red), PE with delivery ≥ 37 weeks (blue) and unaffected pregnancies (black) according to gestational age at time of blood sampling. Diamonds and solid lines represent routine blood samples, circles and dashed lines represent PRESIDE samples. The right‐hand y‐axis shows the effect size in SDs.

The prevalence of preterm PE was 0.7% (62/8386) in the population of women with a routine cFTS blood sample. Based on routine blood sample, pregnancies that developed preterm PE had PAPP‐A MoM values of 0.78 (95% CI, 0.67–0.90) before 10 weeks, 0.80 (95% CI, 0.58–1.10) at 10 weeks and 0.64 (95% CI, 0.53–0.78) at 11–14 weeks. In pregnancies that developed term PE, there appeared to be a minor difference in PAPP‐A levels compared with those of unaffected pregnancies if routine blood samples were collected before 11 weeks (PAPP‐A MoM, 0.98 (95% CI, 0.89–1.09) before 10 weeks and 0.92 (95% CI, 0.80–1.06) at 10 weeks). PAPP‐A levels at 11–14 weeks were lower in pregnancies that developed term PE (PAPP‐A MoM, 0.81 (95% CI, 0.71–0.93)). PAPP‐A was also measured in the PRESIDE samples collected at 11–14 weeks (*n* = 8156). Figure 2 shows the distribution of PAPP‐A MoM in the routine samples and the PRESIDE samples for women with preterm PE, term PE and those with an unaffected pregnancy. PlGF was also measured in these samples. Figure S2 shows the standardized effect size (*Z*‐score) for PAPP‐A MoM and PlGF MoM from routine samples at 8–14 weeks' gestation for women with preterm and term PE as well as for those with an unaffected pregnancy.

**Figure 3:**
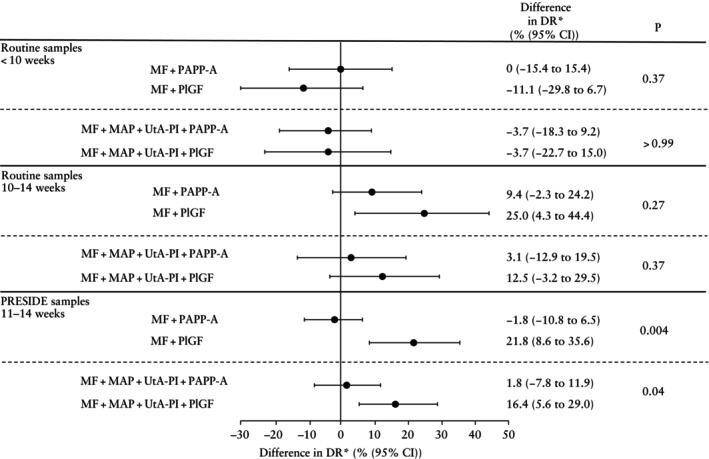
Forest plot showing difference in detection rate (DR), when combining maternal factors (MF) alone or MF, mean arterial pressure (MAP) and uterine artery pulsatility index (UtA‐PI) with either pregnancy‐associated plasma protein‐A (PAPP‐A) or placental growth factor (PlGF) in routine blood samples collected at 8–14 weeks' gestation and in PRESIDE blood samples collected at 11–14 weeks' gestation. *Compared with MF alone or MF + MAP + UtA‐PI without addition of PAPP‐A or PlGF.

The benefit of including either PAPP‐A or PlGF with maternal factors was examined subsequently in terms of the detection rate at a 10% SPR in women with routine samples collected before 10 weeks and at 10–14 weeks, with complete data for risk calculation, including maternal factors, MAP and UtA‐PI (*n* = 8157) (Table 2). For participants with routine blood samples collected before 10 weeks, the inclusion of PAPP‐A or PlGF with maternal factors did not improve the detection rate for preterm PE (Figure 3). Likewise, neither the inclusion of PAPP‐A nor that of PlGF improved the detection rate when combined with maternal factors, MAP and UtA‐PI, in routine samples collected before 10 weeks. In routine samples collected at 10–14 weeks, PAPP‐A increased the detection rate only slightly (and non‐significantly) when combined with maternal factors alone, and the increase in detection rate was even smaller when combined with maternal factors, MAP and UtA‐PI. However, PlGF increased the detection rate significantly when combined with maternal factors, in routine samples collected at 10–14 weeks, from 31.3% (95% CI, 16.1–50.0%) to 56.3% (95% CI, 37.7–73.6%), at a 10% SPR. Furthermore, the detection rate increased from 56.3% (95% CI, 37.7–73.6%) to 68.8% (95% CI, 50.0–83.9%) when maternal factors, MAP and UtA‐PI were combined with PlGF at 10–14 weeks.

**Table 2:** Performance of placental growth factor (PlGF) and pregnancy‐associated plasma protein‐A (PAPP‐A) when added to base test for detection of preterm pre‐eclampsia (PE) (delivery < 37 weeks) in 8157 women with routine blood sample at 8–14 weeks and in 8156 women with PRESIDE sample at 11–14 weeks*

			Addition of PAPP‐A			Addition of PlGF			PlGF *vs* PAPP‐A	
Base test	Preterm PE cases (*n*)	DR of base test†	DR of base test + PAPP‐A	DR increase over base test	*P*	DR of base test + PlGF	DR increase over base test	*P*	Difference in DR	*P*
*Before 10 weeks (routine samples)*										
MF	27	29.6 (13.8–50.2)	29.6 (13.8–50.2)	0 (–15.4 to 15.4)	> 0.99	18.5 (6.3–38.1)	–11.1 (–29.8 to 6.7)	0.37	–11.1 (–29.8 to 6.7)	0.37
MF + MAP + UtA‐PI	27	48.1 (28.7–68.1)	44.4 (25.5–64.7)	–3.7 (–18.3 to 9.2)	> 0.99	44.4 (25.5–64.7)	–3.7 (–22.7 to 15.0)	> 0.99	0 (–20 to 20)	> 0.99
*10–14 weeks (routine samples)*										
MF	32	31.3 (16.1–50.0)	40.6 (23.7–59.4)	9.4 (–2.3 to 24.2)	0.24	56.3 (37.7–73.6)	25.0 (4.3 to 44.4)	0.04	15.6 (–6.9 to 36.8)	0.27
MF + MAP + UtA‐PI	32	56.3 (37.7–73.6)	59.4 (40.6–76.3)	3.1 (–12.9 to 19.5)	> 0.99	68.8 (50.0–83.9)	12.5 (–3.2 to 29.5)	0.22	9.4 (–6.0 to 26.0)	0.37
*11–14 weeks (PRESIDE samples)*										
MF	55	36.4 (23.8–50.4)	34.5 (22.2–48.6)	–1.8 (–10.8 to 6.5)	> 0.99	58.2 (44.1–71.3)	21.8 (8.6 to 35.6)	0.006	23.6 (10.1 to 37.6)	0.004
MF + MAP + UtA‐PI	55	50.9 (37.1–64.6)	52.7 (38.8–66.3)	1.8 (–7.8 to 11.9)	> 0.99	67.3 (53.3–79.3)	16.4 (5.6 to 29.0)	0.02	14.5 (2.6 to 27.6)	0.04

Data are given as % (95% CI), unless stated otherwise.

* Only women with available information on maternal factors (MF), uterine artery pulsatility index (UtA‐PI) and mean arterial pressure (MAP) were included.

† At a fixed 10% screen‐positive rate.

MF include family history of PE, smoking during pregnancy, previous PE, chronic hypertension, Type‐I or ‐II diabetes mellitus, systemic lupus erythematosus, antiphospholipid syndrome, ethnicity, parity and mode of conception.

DR, detection rate.

When examining measurements from blood samples obtained as part of the PRESIDE study, which were all collected at 11–14 weeks (*n* = 8156), we found that the inclusion of PAPP‐A did not significantly increase the detection rate when combined with maternal factors alone or with maternal factors, MAP and UtA‐PI. However, PlGF increased the detection rate significantly both when combined with maternal factors alone (from 36.4% (95% CI, 23.8–50.4%) to 58.2% (95% CI, 44.1–71.3%)) and when combined with maternal factors, MAP and UtA‐PI (from 50.9% (95% CI, 37.1–64.6%) to 67.3% (95% CI, 53.3–79.3%)) (Table 2, Figure 3).

## DISCUSSION

The main finding of this study evaluating the value of PAPP‐A in first‐trimester screening for PE is that the timing of blood sampling did not significantly alter the detection rate for preterm PE. In contrast, the addition of PlGF to maternal factors increased the detection rate for preterm PE by 25.0% (95% CI, 4.3–44.4%) at a 10% SPR at 10–14 weeks in comparison with maternal factors alone. Furthermore, the addition of PlGF to maternal factors, MAP and UtA‐PI increased the detection rate by 16.4% (95% CI, 5.6–29.0%) at a 10% SPR at 11–14 weeks in comparison with the model combining maternal factors, MAP and UtA‐PI alone.

Our finding that PlGF has a greater value than does PAPP‐A in first‐trimester screening for preterm PE is supported by the recent study of Cuenca‐Gómez *et al*.^28^ in a population of 10 110 women with a singleton pregnancy, with a similar incidence of preterm PE of 0.7%, in which blood samples were collected at 11–14 weeks' gestation. Conversely, in a case–cohort study including 1094 women, Noël *et al*.^29^ found a similar sensitivity of combined screening for preterm PE using PAPP‐A (46.7% at a 10% SPR) compared with PlGF (51.7% at a 10% SPR). Notably, the sensitivity reported by Noël *et al*. was much lower than that reported in other studies, probably because the sample size was smaller, and the authors utilized a risk cut‐off of 1 in 50^29^. Only a few previous studies have examined PAPP‐A and PlGF in blood samples collected before 11 weeks in the first‐trimester prediction of preterm PE. Mendoza *et al*.^13^ assessed PAPP‐A and PlGF before 11 weeks in 1675 women and at or after 11 weeks in 966 women. This study did not demonstrate a difference in the area under the receiver‐operating‐characteristics curves when the biochemical markers were assessed before 11 weeks compared with after 11 weeks, and there were no evident differences between the performance of PAPP‐A and that of PlGF. However, with a total of 30 women with preterm PE (16 women with samples collected at 8 + 0 to 10 + 6 weeks and 14 women with samples collected at 11 + 0 to 13 + 6 weeks) in the study, it is difficult to draw any final conclusions owing to insufficient precision. Serra *et al*.^14^ assessed the performance of PAPP‐A and PlGF in 6893 women with blood samples collected between 8 + 0 and 13 + 6 weeks and found that only the addition of PlGF improved the performance of their multivariate Gaussian distribution model, which included maternal factors, MAP and UtA‐PI. It is interesting to note that the biochemical markers were measured at 8–10 weeks' gestation in most cases (83%) in their study^30^.

It has been shown that PlGF MoM is also lower in pregnancies with trisomy 21^31^, and although the impact of PlGF on the overall screening performance for trisomy 21 has been found to be low, it has been suggested that a further improvement in screening for trisomy 21 could be an added benefit of combining first‐trimester screening for aneuploidy and PE using PlGF^14^, ^31^.

A main strength of this study is the large sample size of nearly 8400 women, with blood samples collected between 8 and 14 weeks' gestation, examining the value of PAPP‐A and PlGF separately in PE screening. The participating women were enrolled prospectively from different hospitals and regions of Denmark, thus the PRESIDE cohort is likely to be representative of the Danish pregnant population as a whole. However, it is important to note that all six hospitals are university hospitals. Our findings are relevant for centers aiming to combine first‐trimester screening for aneuploidy and PE detection, particularly in countries with existing first‐trimester screening for aneuploidy similar to that in Denmark. Our study has demonstrated that there is no evidence to support the use of PAPP‐A or PlGF in first‐trimester PE screening when measured before 10 weeks' gestation. It may be considered a limitation of our study that, despite the large sample size, it included a relatively low number of women with preterm PE in each of the gestational‐age windows examined. However, the additional blood sample collected as part of the PRESIDE study at 11–14 weeks validated our finding of PlGF as the most important of the two biochemical markers in first‐trimester screening for PE.

In conclusion, PAPP‐A has limited value in first‐trimester screening for PE, whereas PlGF adds significantly to the detection rate of preterm PE at 10–14 weeks' gestation.

## Supporting information


**Figure S1** Venn diagram showing number of women with a routine blood sample collected at 8–14 weeks' gestation and/or a PRESIDE blood sample collected at 11–14 weeks.
**Figure S2** Distribution of pregnancy‐associated plasma protein‐A (PAPP‐A) and placental growth factor (PlGF) multiples of the median (MoM) values (as *Z*‐scores) in pregnancies with pre‐eclampsia (PE) with delivery < 37 weeks (red), PE with delivery ≥ 37 weeks (blue) and unaffected pregnancies (black) by gestational age at time of blood sampling (median with 95% CI). Diamonds and solid lines represent PlGF, circles and dashed lines represent PAPP‐A.

## Data Availability

The data that support the findings of this study are available on request from the corresponding author. The data are not publicly available due to privacy or ethical restrictions.
