# Supplementary material for: Screening for pre‐eclampsia using pregnancy‐associated plasma protein‐A or placental growth factor measurements in blood samples collected at 8–14 weeks' gestation
Source: Ultrasound Obstet Gynecol. 2025 Mar 24;65(5):567–74. doi: 10.1002/uog.29204 (PMC12047683; doi:10.1002/uog.29204)
Supplement: Supplementary file 1 — Figure S1 Venn diagram showing number of women with a routine blood sample collected at 8–14 weeks' gestation and/or a PRESIDE blood sample collected at 11–14 weeks. Figure S2 Distribution of pregnancy‐associated plasma protein‐A (PAPP‐A) and placental growth factor (PlGF) multiples of the median (MoM) values (as Z‐scores) in pregnancies with pre‐eclampsia (PE) with delivery < 37 weeks (red), PE with delivery ≥ 37 weeks (blue) and unaffected pregnancies (black) by gestational age at time of blood sampling (median with 95% CI). Diamonds and solid lines represent PlGF, circles and dashed lines represent PAPP‐A. [file UOG-65-567-s001.docx]

**Supplementary Figure 1** Venn diagram showing the number of women with a routine blood sample at 8–14 weeks' gestation and/or a PRESIDE blood sample at 11–14 weeks.


**Supplementary Figure 2** Distribution of pregnancy-associated plasma protein-A (PAPP-A) and placental growth factor (PlGF) multiples of the median (MoM) values (as *Z*-scores) in pregnancies with pre-eclampsia < 37 weeks (red), ≥ 37 weeks (blue) and unaffected pregnancies (black) by gestational age at time of blood sampling (median with 95% CI). Diamonds and solid lines represent PlGF, circles and dashed lines represent PAPP-A.
